# Structure-Based Drug Discovery for Invasive Lobular Carcinoma: Druggable Targets and Translational Opportunities

**DOI:** 10.3390/cancers18213413

**Published:** 2026-10-22

**Authors:** Vincent A. Obakachi, Evans E. Ojo

**Affiliations:** DUT-DSTI Space Science & CNS Research Centre, Durban University of Technology, Steve Biko Campus, S7, Level 0, Durban 4000, South Africa

**Keywords:** invasive lobular carcinoma, structure-based drug discovery, *CDH1*, E-cadherin, PI3K/AKT/mTOR, FGFR1, ROS1, YAP/TEAD, precision oncology, artificial intelligence

## Abstract

Background: Invasive lobular carcinoma (ILC) is the second most common histological subtype of breast cancer and is characterized by the loss of E-cadherin resulting from alterations in the *CDH1* gene. Despite accounting for approximately 10–15% of breast cancers, ILC remains understudied compared with invasive ductal carcinoma and presents unique challenges related to diagnosis, endocrine resistance, metastatic progression, and treatment. Recent molecular studies have revealed distinct genomic alterations and signaling dependencies that may provide novel therapeutic opportunities. Methods: This review summarizes current knowledge of the molecular landscape of ILC and critically examines established and emerging therapeutic targets, including PI3K/AKT/mTOR, FGFR1, ROS1, SRC/FAK, Hippo–YAP/TEAD, and estrogen receptor signaling pathways. The application of structure-based drug discovery (SBDD) approaches, including molecular docking, pharmacophore modeling, virtual screening, molecular dynamics simulations, binding free-energy calculations, and artificial intelligence-assisted methodologies, is discussed in the context of ILC drug discovery. Translational opportunities involving natural products, drug repurposing, combination therapies, and precision oncology are also evaluated. Results: Accumulating evidence indicates that *CDH1* loss creates unique molecular vulnerabilities that can be therapeutically exploited. Several signaling pathways involved in tumor growth, survival, invasion, and endocrine resistance have emerged as promising therapeutic targets. Advances in structural biology, computational chemistry, and artificial intelligence have expanded opportunities for rational drug design and target prioritization. Furthermore, drug repurposing strategies, natural product-derived compounds, and multi-target therapeutic approaches offer additional avenues for accelerating the development of ILC-specific treatments. Conclusions: ILC possesses a distinct molecular architecture that supports the development of precision therapeutic strategies beyond those traditionally applied to other breast cancer subtypes. The integration of structural biology, SBDD, artificial intelligence, and translational oncology provides a promising framework for identifying and optimizing therapies targeting *CDH1*-loss-associated vulnerabilities. Continued efforts combining computational discovery with experimental validation are expected to accelerate the development of personalized treatment strategies and improve clinical outcomes for patients with ILC.

## Simple Summary

Invasive lobular carcinoma (ILC) is the second most common subtype of breast cancer and differs from other forms of breast cancer in its biology, clinical behavior, and treatment response. Although significant advances have been made in understanding ILC, effective targeted therapies specifically developed for this disease remain limited. Recent studies have identified several molecular vulnerabilities associated with ILC, including abnormalities in cell adhesion, growth signaling, and mechanisms of therapeutic resistance. At the same time, advances in structural biology, artificial intelligence, and computational drug design have created new opportunities for discovering more effective therapies. This review summarizes the molecular features of ILC, highlights important therapeutic targets, discusses how structure-based drug discovery approaches can accelerate the development of new treatments, and explores future opportunities involving precision oncology, drug repurposing, natural products, and artificial intelligence-assisted drug discovery. These advances may contribute to the development of more personalized and effective treatment strategies for patients with ILC.

## 1. Introduction

Breast cancer remains the most frequently diagnosed malignancy and one of the leading causes of cancer-related mortality among women worldwide. Despite substantial advances in screening, molecular characterization, and targeted therapeutics, breast cancer continues to exhibit remarkable biological and clinical heterogeneity, necessitating subtype-specific therapeutic strategies [1,2,3]. Historically, most research efforts have focused on invasive ductal carcinoma (IDC), the predominant histological subtype. In contrast, invasive lobular carcinoma (ILC), which accounts for approximately 10–15% of all breast cancers, has received comparatively less attention despite its distinctive molecular features, metastatic behavior, and therapeutic challenges [4,5,6,7].

ILC is now recognized as a biologically distinct breast cancer subtype rather than merely a histological variant of IDC. Unlike invasive ductal carcinoma, ILC is characterized by near-universal E-cadherin loss, unique metastatic dissemination patterns, and distinct signaling dependencies that may create subtype-specific therapeutic vulnerabilities [4,8]. The defining hallmark of ILC is the loss of E-cadherin, a transmembrane cell adhesion protein encoded by the *CDH1* gene. Loss-of-function mutations, deletions, or epigenetic silencing of *CDH1* result in disruption of adherens junctions, leading to the characteristic discohesive growth pattern and single-file infiltration observed in ILC tumors [9,10,11]. This unique morphology contributes to diagnostic difficulties, as ILC frequently lacks the palpable masses and radiographic features commonly associated with IDC, often resulting in delayed detection and underestimation of tumor burden [5,12].

Beyond morphological differences, comprehensive genomic analyses have revealed a distinctive molecular landscape in ILC. Large-scale sequencing studies have identified recurrent alterations in genes involved in cell adhesion, hormone signaling, chromatin remodeling, and growth factor pathways. In addition to *CDH1* alterations, frequent mutations have been reported in *PIK3CA*, *ERBB2*, *ESR1*, *FOXA1*, *TBX3*, and *PTEN*, among others [10,13,14,15]. These genomic features contribute to unique transcriptional programs, altered signaling networks, and therapeutic vulnerabilities that differ substantially from those of IDC. Consequently, there is increasing recognition that treatment approaches optimized for IDC may not adequately address the biological complexity of ILC [6,14].

Clinically, the majority of ILC tumors are estrogen receptor-positive (ER+) and HER2-negative, making endocrine therapy a cornerstone of treatment. Nevertheless, a significant proportion of patients eventually develop endocrine resistance, leading to disease progression and late recurrence [6,16]. Several studies suggest that ILC exhibits distinct mechanisms of endocrine adaptation, including alterations in *ESR1* signaling, *FOXA1*-mediated chromatin remodeling, growth factor receptor activation, and dysregulation of the PI3K/AKT pathway [14,16,17]. These observations highlight the urgent need to identify novel therapeutic targets that can overcome resistance mechanisms and improve patient outcomes.

Recent advances in molecular oncology have shifted attention toward identifying actionable vulnerabilities in ILC. Among the most promising opportunities are pathways associated with PI3K/AKT/mTOR signaling, *FGFR1* amplification, SRC and focal adhesion kinase (FAK) signaling, *ROS1*-associated synthetic lethal interactions, and the Hippo–YAP/TEAD pathway [6,8,18,19,20]. Notably, several emerging targets appear to be linked directly or indirectly to *CDH1* loss, suggesting that the defining molecular alteration of ILC may create exploitable therapeutic dependencies. The identification of these vulnerabilities has generated significant interest in developing targeted therapies tailored to the biological context of ILC.

The growing availability of high-resolution protein structures, cryo-electron microscopy data, and AI-predicted protein models has transformed the landscape of modern drug discovery. Structure-based drug discovery (SBDD) integrates structural biology, computational chemistry, medicinal chemistry, and experimental pharmacology to accelerate the identification and optimization of therapeutic candidates [21,22]. Techniques such as molecular docking, pharmacophore modeling, molecular dynamics simulations, free-energy calculations, and machine learning-assisted design have become essential components of contemporary drug discovery pipelines. These approaches offer significant advantages for ILC research, particularly for emerging targets that remain underexplored experimentally.

Despite growing interest in ILC biology and therapeutic development, there remains a lack of reviews that specifically focus on applying structure-based drug discovery strategies to ILC-associated targets. A comprehensive evaluation of druggable vulnerabilities, available structural resources, computational methodologies, and translational opportunities is, therefore, timely and warranted. This review provides an overview of the molecular basis of ILC, discusses established and emerging therapeutic targets, examines the role of structure-based drug discovery approaches in lead identification and optimization, and highlights future directions for the development of precision therapeutics against this challenging breast cancer subtype. The overall molecular framework of ILC, including *CDH1* loss, recurrent genomic alterations, dysregulated signaling pathways, and major opportunities for therapeutic intervention and structure-based drug discovery, is summarized in Figure 1.

## 2. Molecular Landscape of Invasive Lobular Carcinoma

### 2.1. CDH1 Loss and E-Cadherin Dysfunction

Invasive lobular carcinoma (ILC) is distinguished from other breast cancer subtypes by the loss of E-cadherin expression, which is widely regarded as its defining molecular hallmark [10,23]. E-cadherin is a calcium-dependent transmembrane glycoprotein encoded by the *CDH1* gene and plays a fundamental role in maintaining epithelial cell polarity, tissue architecture, and intercellular adhesion through adherens junctions [24]. In normal mammary epithelium, E-cadherin interacts with catenins to establish stable cell–cell contacts and to regulate intracellular signaling pathways that govern proliferation, differentiation, and survival [24,25].

Genetic inactivation of *CDH1* occurs through diverse mechanisms, including truncating mutations, loss of heterozygosity, chromosomal deletions, and epigenetic silencing. The loss of functional E-cadherin disrupts cell adhesion and promotes the characteristic discohesive growth pattern observed in ILC, where tumor cells infiltrate the surrounding stroma in single-file arrangements [23,26]. This distinctive histopathological phenotype contributes to the diffuse nature of ILC lesions and partly explains the diagnostic challenges associated with the disease.

Beyond its structural role, E-cadherin loss has profound biological consequences that extend to multiple oncogenic pathways. Disruption of adherens junctions alters cytoskeletal organization, mechanotransduction, cell migration, and growth factor receptor signaling [25,27]. Several studies have demonstrated that *CDH1*-deficient tumors exhibit increased activation of pathways associated with cell survival, invasion, and therapeutic resistance, thereby creating unique molecular dependencies that may be therapeutically exploitable [18,27]. Consequently, *CDH1* loss is increasingly viewed not only as a diagnostic marker of ILC but also as a source of targetable vulnerabilities.

### 2.2. Recurrent Genomic Alterations in Invasive Lobular Carcinoma

Comprehensive genomic studies have revealed that ILC possesses a molecular landscape distinct from that of invasive ductal carcinoma. In addition to *CDH1* alterations, recurrent mutations have been identified in genes regulating hormone signaling, chromatin dynamics, receptor tyrosine kinase activity, and intracellular signaling cascades [10,15]. Among the most frequently altered genes is *PIK3CA*, which encodes the catalytic subunit of phosphatidylinositol-3-kinase (PI3K). Activating mutations in *PIK3CA* occur in a substantial proportion of ILC cases and contribute to aberrant activation of the PI3K/AKT/mTOR signaling pathway, promoting cellular growth, proliferation, and survival [10,15]. The clinical relevance of this pathway has been reinforced by the development of selective PI3K inhibitors and the growing interest in combination strategies targeting endocrine resistance. Additional genomic events include *ERBB2* (HER2) mutations and amplifications, which occur in a subset of ILC tumors despite the relatively low frequency of HER2 overexpression compared with that in IDC [28]. Activating *ERBB2* mutations may provide actionable therapeutic opportunities through HER2-targeted agents and antibody-drug conjugates. Alterations in *ESR1*, particularly acquired mutations emerging during endocrine therapy, represent another important feature of advanced hormone receptor-positive ILC [14]. These mutations often confer ligand-independent activation of the estrogen receptor and contribute significantly to therapeutic resistance, disease progression, and recurrence.

Recent genomic investigations have also highlighted frequent alterations involving the transcription factor *FOXA1*, a key regulator of estrogen receptor signaling and chromatin accessibility [29]. *FOXA1* mutations appear enriched in ILC relative to IDC and are associated with distinct transcriptional programs that may promote endocrine adaptation and tumor progression. Similarly, recurrent mutations in *TBX3*, a transcription factor involved in developmental pathways and cell differentiation, have been identified as characteristic molecular features of ILC [15]. Although several genomic alterations identified in ILC are also observed in invasive ductal carcinoma (IDC), important differences exist in both frequency and biological context. Unlike IDC, ILC is characterized by near-universal loss of E-cadherin expression resulting from *CDH1* alterations and shows relative enrichment for alterations involving *FOXA1* and *TBX3*. Furthermore, ILC exhibits distinct patterns of endocrine adaptation, metastatic dissemination, and signaling dependencies associated with loss of cell–cell adhesion and altered mechanotransduction. These biological differences contribute to the emergence of therapeutic vulnerabilities that may be particularly relevant to ILC and support the development of subtype-specific precision therapeutic strategies [10,23,24,25]. Collectively, these genomic alterations suggest that ILC is driven by complex interactions among hormone signaling pathways, receptor tyrosine kinase networks, and transcriptional regulatory mechanisms. Understanding these alterations is essential for identifying candidate therapeutic targets and prioritizing drug discovery efforts. The major recurrent genomic alterations identified in ILC, together with their biological functions, functional consequences, therapeutic relevance, and representative intervention strategies, are summarized in Table 1.

### 2.3. Signaling Pathways Driving ILC Progression

The molecular consequences of *CDH1* loss extend beyond individual gene mutations and involve widespread remodeling of cellular signaling networks. Several interconnected pathways have emerged as key drivers of ILC progression and therapeutic resistance. Among these, the PI3K/AKT/mTOR pathway is one of the most extensively studied. Activation of this pathway occurs through *PIK3CA* mutations, *PTEN* loss, growth factor receptor signaling, and adaptive mechanisms associated with endocrine resistance [30,32]. Aberrant PI3K signaling contributes to increased cell survival, proliferation, and metastatic potential, making it a major therapeutic target.

The FGFR signaling pathway has also attracted significant attention. Amplification of *FGFR1* has been associated with endocrine resistance, disease progression, and poor clinical outcomes in hormone receptor-positive breast cancers, including subsets of ILC [34]. Activation of FGFR signaling promotes downstream activation of the MAPK and PI3K pathways, creating opportunities for therapeutic targeting with selective FGFR inhibitors.

Increasing evidence further implicates SRC and focal adhesion kinase (FAK) signaling in the invasive and migratory phenotype of *CDH1*-deficient tumors [31]. Alterations in cell adhesion resulting from E-cadherin loss can enhance SRC/FAK activity, leading to cytoskeletal remodeling, increased motility, and metastatic dissemination. These observations have stimulated interest in SRC and FAK inhibition as potential therapeutic approaches for ILC.

Recent studies have also highlighted the role of the Hippo–YAP/TEAD signaling pathway, which is a critical regulator of mechanotransduction, proliferation, and tissue homeostasis [33]. Disruption of cell–cell adhesion following E-cadherin loss may promote YAP/TAZ activation, thereby enhancing tumor cell survival and transcriptional reprogramming. Given the growing success of structure-based approaches targeting YAP-TEAD interactions, this pathway represents an attractive frontier for future drug discovery efforts.

Another promising area involves synthetic lethal vulnerabilities associated with *CDH1* loss. Functional genomic screens have identified several pathways and targets that become essential in the absence of E-cadherin, including ROS1-related signaling networks and growth factor receptor-dependent mechanisms [8,18]. Exploiting such vulnerabilities may enable the development of highly selective therapeutic strategies for ILC while minimizing toxicity to normal tissues.

Taken together, the molecular landscape of ILC reveals a complex network of genomic alterations and signaling dependencies that collectively drive tumor initiation, progression, metastasis, and therapeutic resistance. These pathways provide a rich source of druggable targets and establish a strong rationale for applying structure-based drug discovery approaches to this disease. As illustrated in Figure 2, *CDH1* loss is a central molecular event that drives multiple signaling dependencies, including the PI3K/AKT/mTOR, *FGFR1*, SRC/FAK, ROS1, and YAP/TEAD pathways, thereby creating several actionable therapeutic vulnerabilities in ILC.

## 3. Druggable Targets in Invasive Lobular Carcinoma

The growing understanding of ILC biology has revealed several actionable molecular targets that may be exploited for therapeutic intervention. Unlike conventional treatment strategies largely extrapolated from invasive ductal carcinoma, emerging evidence suggests that ILC harbors distinct signaling dependencies associated with *CDH1* loss, endocrine adaptation, and activation of survival pathways. Consequently, the identification and validation of druggable targets have become central to the development of precision therapies for ILC. The current landscape of established and emerging therapeutic targets in ILC, including their associated signaling pathways, biological rationale, representative inhibitors, and clinical development status, is summarized in Table 2.

### 3.1. PI3K/AKT/mTOR Signaling Pathway

The PI3K/AKT/mTOR pathway represents one of the most clinically validated therapeutic targets in ILC and is among the most frequently dysregulated signaling networks in this disease. Activating mutations in *PIK3CA* occur in approximately 40–50% of cases and contribute to enhanced cellular proliferation, metabolic adaptation, survival, and therapeutic resistance [13,35]. Additional alterations involving *PTEN* loss and AKT activation further reinforce the importance of this pathway in disease progression [36]. Clinical validation of PI3K signaling as a therapeutic target was established through the development of alpelisib, a selective PI3Kα inhibitor that demonstrated significant clinical benefit in hormone receptor-positive, *PIK3CA*-mutated breast cancer [27]. Because *PIK3CA* mutations are particularly common in ILC, this pathway is among the most clinically actionable targets currently available [13,35]. Despite encouraging clinical outcomes, therapeutic resistance frequently emerges through compensatory signaling mechanisms involving AKT, mTOR, and receptor tyrosine kinases [30,36]. Structure-based drug discovery approaches are therefore increasingly focused on next-generation PI3K inhibitors and combination therapies that simultaneously suppress multiple nodes within this signaling network.

### 3.2. FGFR Signaling

Aberrant fibroblast growth factor receptor (FGFR) signaling has emerged as another important driver of endocrine-resistant breast cancer. Amplification of *FGFR1* is enriched in subsets of hormone receptor-positive breast cancers and has been associated with poor prognosis, disease progression, and reduced responsiveness to endocrine therapies [34,37]. Activation of FGFR signaling promotes downstream MAPK, PI3K/AKT, and STAT pathways, thereby enhancing cell proliferation and survival [37]. Importantly, multiple crystal structures of FGFR kinases are available in the Protein Data Bank, making the pathway highly amenable to structure-based drug design [38]. Several FGFR inhibitors, including erdafitinib, pemigatinib, futibatinib, and infigratinib, have demonstrated encouraging activity across FGFR-driven malignancies [38]. Although their clinical application in ILC remains underexplored, FGFR1 should currently be regarded as an emerging but increasingly validated therapeutic target, particularly in endocrine-resistant disease, where growing biological and translational evidence supports its relevance.

### 3.3. ROS1 and Synthetic-Lethal Vulnerabilities

Synthetic lethality represents a particularly attractive therapeutic strategy for ILC because it exploits molecular dependencies arising directly from *CDH1* loss. Rather than targeting E-cadherin itself, synthetic-lethal approaches identify pathways that become essential only in *CDH1*-deficient cells [39]. One of the most notable discoveries in this area involves ROS1, a receptor tyrosine kinase identified as a selective vulnerability in E-cadherin-deficient tumors [15,19]. Preclinical studies have demonstrated that pharmacological inhibition of ROS1 selectively impairs the viability of *CDH1*-deficient cancer cells while exerting comparatively limited effects on *CDH*1-proficient counterparts [15]. The synthetic-lethal relationship between *CDH1* loss and *ROS1* signaling provides a compelling rationale for precision therapeutic strategies targeting ILC. Despite its strong biological rationale, ROS1 should presently be considered an emerging ILC-associated therapeutic vulnerability rather than a clinically validated target, as most supporting evidence remains pre-clinical and requires further validation in ILC-specific models and clinical settings. From a drug discovery perspective, ROS1 is especially attractive because numerous kinase structures and clinically approved inhibitors are already available, facilitating rapid translational development [42,43].

### 3.4. SRC and FAK Signaling

Disruption of E-cadherin-mediated adhesion significantly alters cellular mechanotransduction and promotes activation of non-receptor tyrosine kinases such as SRC and focal adhesion kinase (FAK) [28,46]. These pathways regulate cytoskeletal organization, migration, invasion, extracellular matrix interactions, and metastatic dissemination. Experimental studies have shown that *CDH1*-deficient cells exhibit enhanced dependence on SRC and FAK signaling for survival and motility [45]. Consequently, inhibition of these kinases has emerged as a promising strategy for limiting metastasis and tumor progression in ILC. However, SRC and FAK currently remain emerging therapeutic opportunities, with much of the available evidence derived from preclinical studies and extrapolation from broader cancer research. Several small-molecule SRC and FAK inhibitors have already entered clinical development, including dasatinib, bosutinib, and defactinib [44]. The availability of high-resolution kinase structures has enabled extensive structure-based optimization efforts to enhance potency, selectivity, and pharmacokinetic properties.

### 3.5. Hippo–YAP/TEAD Signaling

The Hippo pathway is a key regulator of tissue homeostasis, organ size control, mechanosensing, and cellular proliferation. Dysregulation of this pathway results in activation of the transcriptional coactivators YAP and TAZ, which subsequently interact with TEAD transcription factors to drive oncogenic gene expression programs [33]. Recent evidence suggests that disruption of cell–cell adhesion resulting from E-cadherin loss promotes YAP/TAZ activation, thereby linking *CDH1* deficiency to Hippo pathway deregulation [47]. Enhanced YAP/TAZ signaling has been associated with increased tumor growth, metastatic potential, stemness, and therapeutic resistance.

Historically, YAP and TEAD were considered difficult therapeutic targets because they function primarily through protein–protein interactions. However, recent advances in structural biology have identified druggable pockets within TEAD proteins and enabled the development of novel small-molecule TEAD inhibitors [48,49]. This progress has transformed the YAP/TEAD complex into one of the most promising emerging therapeutic opportunities in ILC. Nevertheless, YAP/TEAD-targeted therapies remain in the early stages of development, and their clinical utility in ILC has yet to be established.

### 3.6. Estrogen Receptor Signaling and Endocrine Resistance

Estrogen receptor (ER) signaling remains a dominant driver of ILC biology, as most tumors are estrogen receptor-positive and initially responsive to endocrine therapy [14,16]. However, prolonged treatment frequently leads to adaptive resistance mediated by *ESR1* mutations, chromatin remodeling, activation of growth factor pathways, and transcriptional reprogramming [14,29]. Mutations within the ligand-binding domain of *ESR1* promote constitutive receptor activation and reduce dependence on estrogen stimulation [14]. In parallel, alterations involving *FOXA1* and associated chromatin regulatory mechanisms contribute to endocrine adaptation and tumor persistence [29]. These observations have stimulated the development of next-generation endocrine therapeutics, including selective estrogen receptor degraders (SERDs), selective estrogen receptor modulators (SERMs), proteolysis-targeting chimeras (PROTACs), and combination regimens incorporating PI3K, CDK4/6, or FGFR inhibitors [14,40]. ER signaling represents a clinically validated therapeutic vulnerability and remains a cornerstone target in ILC, particularly because most tumors are hormone receptor-positive and initially responsive to endocrine therapies; however, durable clinical responses will likely require combination approaches to overcome adaptive resistance mechanisms. The interconnected signaling landscape and major druggable targets discussed throughout this section are illustrated in Figure 3, which highlights the extensive crosstalk among the PI3K/AKT/mTOR, FGFR1, ROS1, SRC/FAK, YAP/TEAD, and estrogen receptor signaling pathways.

### 3.7. Evidence Hierarchy and Translational Readiness of ILC Therapeutic Targets

The therapeutic targets discussed in this review differ substantially in the strength of evidence supporting their relevance to ILC. PI3K/AKT/mTOR signaling and ER/ESR1-directed therapies represent the most clinically validated therapeutic vulnerabilities, supported by extensive biological, translational, and clinical evidence in hormone receptor-positive breast cancer, including ILC, and have already yielded approved targeted therapies for appropriately selected patient populations [10,11,13,14,15,26,34,50,51].

FGFR1 may be considered an emerging yet increasingly validated therapeutic target, as accumulating evidence links *FGFR1* amplification and activation of signaling to endocrine resistance, disease progression, and poor clinical outcomes in subsets of ILC and hormone receptor-positive breast cancer [14,17,31,33,52].

In contrast, ROS1, SRC/FAK, and YAP/TEAD currently remain emerging therapeutic opportunities. Although these pathways are supported by compelling biological rationale, mechanistic studies, and preclinical findings, additional validation in ILC-specific experimental systems and clinical settings remains necessary before their therapeutic utility can be fully established [18,30,36,37,38,39,41,42,43].

Similarly, several drug repurposing strategies, natural product-derived compounds, and multi-target therapeutic approaches should presently be regarded as hypothesis-generating opportunities rather than established therapeutic interventions, as much of the available supporting evidence is derived from broader breast cancer research, pan-cancer studies, or computational investigations rather than direct ILC-specific validation [11,53,54]. Recognition of these differences in evidentiary support is essential for prioritizing future translational research and guiding structure-based drug discovery efforts in ILC.

## 4. Structure-Based Drug Discovery Strategies for Invasive Lobular Carcinoma

The growing availability of high-resolution protein structures, advancements in computational chemistry, and the emergence of artificial intelligence have transformed the drug discovery landscape. In ILC, where several signaling pathways and molecular dependencies have been identified yet remain insufficiently exploited therapeutically, structure-based drug discovery (SBDD) offers a powerful framework to accelerate lead identification, optimization, and translational development. By integrating structural biology with computational and experimental approaches, SBDD enables rational targeting of both established oncogenic drivers and emerging vulnerabilities associated with CDH1 loss. A typical structure-based drug discovery workflow applicable to ILC therapeutic discovery is illustrated in Figure 4. The workflow integrates target identification, structural characterization, virtual screening, molecular docking, molecular dynamics simulations, binding free-energy calculations, lead optimization, and experimental validation.

### 4.1. Structural Biology Resources for ILC Drug Discovery

Successful implementation of SBDD relies on the availability of accurate three-dimensional protein structures. Multiple ILC-relevant targets currently possess experimentally determined structures deposited in the Protein Data Bank (PDB), including PI3Kα, AKT, mTOR, FGFR1, HER2, ROS1, SRC, FAK, and ESR1 [55,56,57]. These structural resources provide detailed information regarding catalytic sites, ligand-binding pockets, allosteric regions, and protein–protein interaction interfaces that can be exploited for therapeutic intervention. Recent advances in cryo-electron microscopy (cryo-EM) have further expanded the repertoire of structurally characterized therapeutic targets, particularly large protein complexes that were previously difficult to resolve using conventional crystallography [58]. Additionally, the development of AlphaFold has dramatically improved access to high-quality structural models for proteins lacking experimentally determined structures, enabling exploration of emerging targets involved in ILC progression [22,59]. Structural information is particularly valuable in ILC because several promising therapeutic opportunities, including YAP/TEAD complexes and synthetic-lethal effectors, remain underexplored experimentally. High-confidence structural models facilitate virtual screening, binding-site identification, mutational analysis, and rational inhibitor design.

Importantly, structure-guided drug discovery has already contributed to the development of therapeutics targeting pathways highly relevant to ILC. Structural characterization of PI3Kα, FGFR1, and ERα has supported the discovery and optimization of clinically relevant inhibitors and endocrine therapies, while the recent identification of druggable lipid-binding pockets within TEAD proteins has accelerated the development of inhibitors targeting the emerging Hippo–YAP pathway [60,61,62,63]. The availability and maturity of structural resources vary considerably across ILC-associated therapeutic targets. While PI3Kα, AKT, mTOR, ESR1, HER2, and FGFR1 have benefited from extensive structure-guided drug discovery efforts, emerging targets such as ROS1, SRC, FAK, and YAP/TEAD are only beginning to be explored in the context of ILC. Recent structural characterization of TEAD proteins has also revealed druggable lipid-binding pockets, providing new opportunities for targeting Hippo–YAP signaling in CDH1-deficient tumors. The current structural landscape and structure-based drug discovery readiness of major ILC-associated targets are summarized in Table 3.

Importantly, although several targets have extensive structural information and clinically validated inhibitors, others, such as ROS1, SRC/FAK, and YAP/TEAD, remain emerging therapeutic opportunities in ILC, with much of the current evidence derived from preclinical studies and extrapolation from broader cancer research.

### 4.2. Molecular Docking in ILC Drug Discovery

Molecular docking remains one of the most widely used computational techniques in early-stage drug discovery. The method predicts the preferred binding orientation of a small molecule within a target protein and estimates the strength of receptor–ligand interactions [21,72,73]. For ILC-associated targets, docking has become an essential tool for prioritizing compounds against kinases such as PI3K, FGFR1, ROS1, SRC, and FAK. The approach enables rapid screening of large chemical libraries while providing detailed insights into molecular recognition, hydrogen-bond networks, hydrophobic contacts, electrostatic interactions, and steric complementarity [72,74,75]. Modern docking protocols increasingly employ consensus-scoring strategies, ensemble docking, and induced-fit approaches to improve predictive accuracy.

Furthermore, integrating docking with experimental validation substantially increases hit identification rates and reduces the time and cost associated with traditional screening campaigns [53]. Despite its utility, docking alone cannot fully capture protein flexibility or solvent effects. Consequently, promising docking hits typically require further refinement using molecular dynamics simulations and free-energy calculations.

### 4.3. Pharmacophore Modeling and Virtual Screening

Pharmacophore modeling represents another valuable component of contemporary SBDD workflows. A pharmacophore describes the essential steric and electronic features required for molecular recognition by a biological target [76]. Both ligand-based and structure-based pharmacophore models have been successfully applied in oncology drug discovery to identify novel inhibitors with desired binding characteristics [77]. In the context of ILC, pharmacophore-guided screening may facilitate the discovery of compounds targeting PI3K, FGFR1, ROS1, and YAP/TEAD signaling pathways.

Virtual screening incorporates pharmacophore models, molecular docking, and cheminformatics filters to computationally evaluate thousands to millions of compounds before experimental testing [21,78]. This strategy significantly enriches hit rates while reducing laboratory costs and resource requirements. The increasing availability of natural-product databases, commercially available libraries, and AI-generated molecular collections further expands the chemical space accessible to virtual screening campaigns targeting ILC-associated targets.

### 4.4. Molecular Dynamics Simulations

While docking provides a static representation of receptor–ligand interactions, molecular dynamics (MD) simulations offer a dynamic description of biomolecular behavior under physiologically relevant conditions [79,80]. MD simulations allow researchers to evaluate

- Complex stability;
- Ligand-binding persistence;
- Protein conformational changes;
- Residue flexibility;
- Solvent interactions;
- Allosteric effects.

For ILC drug discovery, MD simulations have become indispensable for validating docking predictions and identifying stable binding modes for candidate therapeutics [81,82,83]. Moreover, MD facilitates characterization of resistance-associated mutations and helps predict how genomic alterations affect target structure and drug binding. Several metrics are routinely employed in simulation analyses, including root-mean-square deviation (RMSD), root-mean-square fluctuation (RMSF), radius of gyration (Rg), solvent-accessible surface area (SASA), and hydrogen-bond occupancy [84,85]. The integration of MD with experimental studies improves confidence in identified lead compounds and provides mechanistic insights that cannot be obtained through docking alone.

### 4.5. Binding Free-Energy Calculations

Binding free-energy calculations constitute an important bridge between docking predictions and experimental validation. Methods such as molecular mechanics/generalized Born surface area (MM/GBSA) and molecular mechanics/Poisson-Boltzmann surface area (MM/PBSA) estimate ligand-binding affinities by combining molecular mechanics energies with solvation effects [86,87]. These approaches have gained widespread acceptance because they provide more reliable estimates of binding strength than docking scores alone while remaining computationally tractable [88,89].

In cancer drug discovery, MM/GBSA analyses are frequently employed after MD simulations to

- Rank lead compounds;
- Identify key interacting residues;
- Evaluate energetic contributions;
- Guide medicinal chemistry optimization.

The application of free-energy calculations is particularly advantageous for ILC-associated kinase targets because subtle structural differences often determine selectivity, potency, and resistance profiles.

#### Representative Applications of Structure-Based Drug Discovery to ILC-Relevant Targets

The practical utility of structure-based drug discovery is illustrated by several successful therapeutic programs targeting highly relevant ILC biology. For example, structure-guided optimization of PI3Kα inhibitors contributed to the development of clinically approved agents such as alpelisib and next-generation inhibitors, including inavolisib, both of which target aberrant PI3K signaling frequently observed in ILC [60,61]. Similarly, structural characterization of FGFR kinase domains facilitated the development of selective FGFR inhibitors, including erdafitinib and pemigatinib, which may be relevant to FGFR1-amplified and endocrine-resistant ILC [62,90].

Structure-based approaches have also played important roles in the development of ER-targeted therapeutics. High-resolution structures of the ERα ligand-binding domain have enabled the rational design and optimization of selective estrogen receptor degraders (SERDs) and modulators used to overcome endocrine resistance in hormone receptor-positive breast cancer [91]. More recently, the identification of druggable palmitoylation pockets within TEAD proteins has accelerated the development of novel Hippo–YAP pathway inhibitors, including VT3989 and related compounds, offering promising opportunities to target mechanotransduction-associated vulnerabilities in CDH1-deficient tumors [70,92].

Together, these examples demonstrate how structural biology and computational modeling can contribute directly to the identification, optimization, and clinical translation of therapies targeting pathways relevant to ILC.

### 4.6. Multi-Target Drug Design Approaches

Cancer progression is driven by interconnected signaling networks rather than isolated molecular pathways. Consequently, inhibition of a single target frequently results in compensatory pathway activation and therapeutic resistance [30]. This challenge is especially relevant in ILC, where endocrine resistance often arises through crosstalk among ER, PI3K, FGFR1, SRC, and growth factor signaling pathways [30]. Multi-target drug design, therefore, represents an attractive strategy for simultaneously modulating multiple disease-driving mechanisms. Structure-based approaches can facilitate the discovery of dual-acting or polypharmacological compounds through

- Multi-target pharmacophore construction;
- Cross-docking analyses;
- Binding-site similarity assessments;
- AI-guided molecular generation.

Several target combinations appear particularly attractive in ILC because they simultaneously target complementary pathways involved in tumor growth, survival, invasion, endocrine resistance, and adaptive signaling responses.

a. PI3K + FGFR1

Simultaneous inhibition of two major drivers of endocrine resistance and compensatory survival signaling.

b. PI3K + ESR1

Combines growth pathway inhibition with suppression of estrogen receptor signaling, a dominant driver of ILC biology.

c. SRC + FAK

Targets complementary regulators of cell adhesion, migration, invasion, and metastatic dissemination.

d. ROS1 + PI3K

Integrates synthetic-lethal targeting associated with CDH1 loss with inhibition of tumor-survival signaling pathways.

e. YAP/TEAD + PI3K

Suppresses mechanotransduction-associated transcriptional programs while simultaneously blocking compensatory growth signaling.

These combinations are supported by the extensive signaling crosstalk that characterizes ILC and may provide greater therapeutic durability than single-target approaches by reducing compensatory pathway activation and adaptive resistance mechanisms. Collectively, advances in structural biology, molecular docking, pharmacophore modeling, MD simulations, free-energy calculations, and multi-target design have established a robust computational framework for accelerating therapeutic discovery in ILC. These tools are increasingly positioned at the center of precision oncology efforts to exploit both established oncogenic drivers and emerging molecular vulnerabilities associated with CDH1 loss. The success of structure-guided therapeutic development targeting PI3Kα, FGFR1, and ERα demonstrates the practical impact of SBDD in modern oncology. Although many of these therapies were initially developed for broader cancer indications, their targets are highly relevant to ILC biology and provide a framework for future efforts to exploit CDH1-loss-associated vulnerabilities, synthetic-lethal interactions, and mechanotransduction-associated pathways unique to ILC [60,61,62,63].

## 5. Translational Opportunities

The successful translation of molecular discoveries into clinically effective therapies remains a major challenge in oncology. In ILC, advances in genomic profiling and target identification have generated numerous opportunities for therapeutic development, yet relatively few ILC-specific drugs have reached clinical implementation. Bridging this gap requires integrating innovative drug discovery strategies, rational combination therapies, drug repurposing approaches, and biologically relevant experimental models. Structure-based drug discovery has the potential to accelerate this transition by facilitating the identification and optimization of compounds targeting both established oncogenic drivers and emerging vulnerabilities associated with *CDH1* loss. The translational pathway linking computational discovery, experimental validation, biomarker development, and clinical implementation in ILC is summarized in Figure 5. This framework highlights how structure-based drug discovery can be integrated with precision oncology approaches to accelerate therapeutic development.

### 5.1. Natural Products and Natural Product-Derived Scaffolds

Natural products have historically served as a rich source of anticancer agents and continue to play a vital role in modern drug discovery. Several clinically approved anticancer drugs, including paclitaxel, vincristine, vinblastine, camptothecin derivatives, and epothilones, originated directly or indirectly from natural sources [93,94].

The structural complexity and chemical diversity of natural products often provide unique pharmacophores that can modulate challenging biological targets. Growing evidence suggests that numerous phytochemicals can influence pathways highly relevant to ILC, including PI3K/AKT/mTOR, FGFR, Src family kinases, and Hippo–YAP signaling [95,96].

Recent advances in computational chemistry have enabled the large-scale virtual screening of natural product libraries against cancer-associated targets. Structure-based approaches combining pharmacophore modeling, molecular docking, molecular dynamics simulations, and free-energy calculations can facilitate the identification of novel natural product-inspired inhibitors with improved potency and selectivity [97].

Furthermore, natural product scaffolds provide attractive starting points for medicinal chemistry optimization and hybrid molecule design. The combination of natural product pharmacophores with synthetic modifications may generate compounds possessing enhanced bioavailability, reduced toxicity, and improved target specificity for ILC-associated pathways. Several natural products and natural product-derived compounds have been reported to modulate signaling pathways relevant to ILC, including PI3K/AKT/mTOR, ER, FGFR1, SRC/FAK, and Hippo–YAP signaling. The inclusion of an evidence-level classification highlights the current translational maturity of each compound and identifies opportunities for future validation in ILC-specific models. Representative examples and their translational relevance are summarized in Table 4.

Importantly, direct evidence supporting the use of many of these compounds in ILC-specific experimental models remains limited. While several natural products demonstrate activity against pathways relevant to ILC biology, much of the available evidence originates from broader breast cancer studies or pan-cancer investigations. Therefore, these agents should currently be regarded as promising hypothesis-generating opportunities rather than established therapeutic strategies for ILC and warrant further evaluation in ILC-specific cellular, organoid, and preclinical models.

### 5.2. Drug Repurposing Opportunities

Drug repurposing has emerged as an increasingly attractive strategy for accelerating the development of cancer therapeutics. By identifying new applications for approved or clinically investigated drugs, repurposing can substantially reduce development timelines, costs, and safety-related risks [110]. Several approved anticancer agents targeting pathways relevant to ILC may offer opportunities for rapid clinical translation. Examples include PI3K inhibitors, FGFR inhibitors, HER2-directed therapeutics, ROS1 inhibitors, and CDK4/6 inhibitors [32,40]. Additionally, drugs originally developed for other malignancies may demonstrate efficacy against ILC-specific molecular vulnerabilities. Structure-based drug repurposing approaches utilize computational screening of approved drug libraries against newly identified targets. Molecular docking and binding free-energy analyses can reveal previously unrecognized target-ligand interactions and generate testable hypotheses for experimental validation [111]. Given the limited number of clinical trials specifically dedicated to ILC, drug repurposing represents a practical and cost-effective approach to rapidly expanding therapeutic options for patients.

### 5.3. Combination Therapy Strategies

Monotherapies targeting individual signaling pathways often yield only transient clinical responses because cancer cells can activate compensatory networks that promote survival and resistance [30]. This challenge is particularly relevant in ILC, where extensive crosstalk exists among estrogen receptor signaling, PI3K/AKT/mTOR, FGFR, SRC/FAK, and growth factor receptor pathways [112]. Consequently, combination therapies are increasingly being investigated to improve treatment durability and overcome resistance mechanisms. Several biologically rational combinations have emerged as particularly promising for ILC:

- ER inhibition + CDK4/6 inhibition;
- ER inhibition + PI3K inhibition;
- PI3K inhibition + FGFR inhibition;
- ROS1 inhibition + endocrine therapy;
- SRC/FAK inhibition + endocrine therapy;
- YAP/TEAD inhibition + PI3K inhibition.

Structure-based approaches can play an important role in identifying synergistic target combinations and supporting the development of multi-target therapeutics. Simultaneous modulation of complementary pathways may improve therapeutic efficacy while minimizing adaptive resistance [113].

### 5.4. Experimental Validation Models

The success of computational drug discovery ultimately depends on experimental validation. Therefore, appropriate biological models are essential for translating in silico predictions into clinically relevant findings.

#### 5.4.1. ILC Cell Line Models

Several established ILC cell lines are widely used for mechanistic studies and therapeutic evaluation, including MDA-MB-134-VI, SUM44PE, MM134, and BCK4. These models recapitulate important aspects of ILC biology, including hormone dependence, CDH1 deficiency, and endocrine responsiveness [114].

#### 5.4.2. Three-Dimensional Organoid Models

Three-dimensional organoid systems have transformed cancer research by providing physiologically relevant models that better preserve tumor architecture and cellular heterogeneity than conventional two-dimensional cultures [115]. Patient-derived organoids can be used to

- Evaluate drug sensitivity;
- Investigate resistance mechanisms;
- Perform biomarker discovery;
- Validate computational predictions.

#### 5.4.3. Patient-Derived Xenografts and Ex Vivo Models

Patient-derived xenografts (PDXs) remain valuable tools for assessing in vivo therapeutic efficacy and pharmacological responses. ILC-specific PDX models have facilitated investigation of endocrine resistance, metastatic dissemination, and target dependency in clinically relevant settings [54]. Together, cell lines, organoids, and PDX systems provide complementary experimental platforms that support translational drug discovery efforts.

### 5.5. Precision Oncology Opportunities

The integration of molecular profiling, computational drug discovery, and functional validation is driving the emergence of precision oncology approaches tailored to individual tumor characteristics. ILC appears particularly suitable for precision medicine because of its distinctive genomic architecture and recurrent signaling dependencies. Comprehensive genomic profiling can facilitate patient stratification based on: *PIK3CA* mutation status, *FGFR1* amplification, *ESR1* mutations, *ERBB2* alterations, and *CDH1*-associated vulnerabilities. Such stratification may enable the selection of targeted therapies that are more likely to benefit specific patient populations while minimizing unnecessary treatment exposure [116]. Future precision oncology strategies will likely combine multi-omics data, artificial intelligence, structure-based drug discovery, and functional screening approaches to identify patient-specific therapeutic vulnerabilities. These advances may ultimately lead to more effective and individualized treatment options for patients with ILC.

## 6. Challenges and Future Perspectives

Despite substantial advances in understanding the biology of invasive lobular carcinoma (ILC), significant challenges continue to impede the development of effective ILC-specific therapeutics. Compared with other breast cancer subtypes, ILC remains relatively underrepresented in both translational research and clinical trials. Overcoming these limitations will require integrating molecular profiling, structure-based drug discovery, artificial intelligence, innovative experimental models, and precision oncology approaches. Future progress will depend on identifying and exploiting the unique biological vulnerabilities associated with *CDH1* loss while developing therapeutics that overcome resistance mechanisms and tumor heterogeneity.

### 6.1. Biological Challenges

One of the primary challenges in ILC research is its considerable molecular and clinical heterogeneity. Although *CDH1* loss represents the defining molecular hallmark of the disease, additional genomic alterations involving *PIK3CA*, *ESR1*, *ERBB2*, *FGFR1*, *FOXA1*, *TBX3*, and other genes contribute to substantial interpatient variability [4,10,15]. Consequently, therapeutic responses often differ between patients despite similar histopathological diagnoses.

Another major challenge is the development of endocrine resistance. While most ILC tumors are initially responsive to anti-estrogen therapies, resistance frequently emerges through mechanisms involving *ESR1* mutations, PI3K pathway activation, growth factor receptor signaling, and transcriptional reprogramming [14,29,30]. These adaptive responses often diminish the long-term effectiveness of endocrine therapies and contribute to disease recurrence.

The unique metastatic behavior of ILC further complicates the development of therapies. Unlike invasive ductal carcinoma, ILC preferentially metastasizes to unusual anatomical sites, including the gastrointestinal tract, peritoneum, ovaries, leptomeninges, and bone, creating significant challenges for disease monitoring and treatment [5,16]. Improved understanding of the molecular determinants underlying these metastatic patterns may reveal novel therapeutic opportunities.

### 6.2. Challenges in Structure-Based Drug Discovery

Although structure-based drug discovery has transformed modern therapeutic development, several limitations remain relevant to ILC-associated targets. One challenge is the incomplete structural characterization of certain emerging therapeutic targets and protein complexes. While high-resolution structures are available for kinases such as PI3K, FGFR1, ROS1, SRC, and FAK, structural information for some transcriptional regulators and signaling complexes remains limited [55,56,57,58,59].

Targeting protein–protein interactions (PPIs) presents an additional difficulty. Many promising ILC-associated vulnerabilities, including components of the Hippo–YAP/TEAD pathway and other transcriptional regulatory complexes, involve large and relatively flat interaction surfaces that have historically been difficult to modulate with conventional small molecules [48,49]. Recent advances have improved the tractability of these targets, but further methodological developments are still required.

Another challenge involves accurately predicting therapeutic efficacy from computational analyses. Molecular docking and free-energy calculations provide valuable insights into ligand-target interactions; however, they cannot fully reproduce the complexity of biological systems. Factors such as tumor microenvironment interactions, pharmacokinetics, protein dynamics, and adaptive signaling responses often influence clinical outcomes and remain difficult to model computationally [72,79,88]. The major biological, computational, translational, and clinical challenges currently limiting structure-based drug discovery in ILC, together with potential mitigation strategies and future opportunities, are summarized in Table 5.

Addressing these challenges will be essential for translating computational discoveries into clinically effective therapeutics and realizing the full potential of precision oncology in ILC.

### 6.3. Opportunities for Precision Oncology

The rapid expansion of multi-omics technologies has created unprecedented opportunities for precision medicine in ILC. Comprehensive genomic, transcriptomic, proteomic, and metabolomic profiling can facilitate identification of patient-specific vulnerabilities and enable more precise therapeutic stratification [116,117]. Future precision oncology approaches may incorporate

- Genomic profiling of actionable mutations;
- Transcriptomic characterization of signaling dependencies;
- Functional validation using organoids and patient-derived models;
- Biomarker-guided treatment selection;
- Real-time monitoring of resistance mechanisms.

Such strategies could enable clinicians to match individual patients with therapies targeting the dominant molecular drivers of their tumors rather than relying solely on histological classification. Furthermore, the identification of synthetic lethal interactions associated with *CDH1* deficiency may provide opportunities for highly selective therapeutic interventions with reduced off-target toxicity [19,52]. This concept is particularly attractive in ILC because it exploits vulnerabilities that arise directly from the disease’s defining genetic alteration.

### 6.4. Artificial Intelligence and Machine Learning in ILC Drug Discovery

Artificial intelligence (AI) and machine learning (ML) are increasingly reshaping the drug discovery process. AI-driven approaches can accelerate target identification, virtual screening, de novo molecular design, toxicity prediction, and lead optimization [119,120]. Within the context of ILC, AI may facilitate

- Identification of novel *CDH1*-associated vulnerabilities;
- Prediction of synthetic-lethal interactions;
- Discovery of multi-target therapeutic agents;
- Optimization of natural product-derived scaffolds;
- Prediction of drug resistance mechanisms;
- Integration of multi-omics datasets for patient stratification.

Recent advances in deep learning-based protein structure prediction, exemplified by AlphaFold, have significantly expanded access to structural information for previously uncharacterized proteins and are expected to have a substantial impact on future oncology drug discovery efforts [22,59]. Generative AI platforms are also increasingly capable of designing novel compounds with desired physicochemical and biological properties, potentially accelerating the discovery of next-generation therapeutics targeting complex ILC-associated pathways [120,121].

### 6.5. Future Research Directions

Several research priorities should be emphasized to accelerate therapeutic development for ILC:

#### 6.5.1. Target Priorities

- *CDH1*-loss-associated synthetic-lethal vulnerabilities;
- ROS1 signaling dependencies;
- FGFR1-driven endocrine resistance;
- SRC/FAK signaling networks;
- Hippo–YAP/TEAD pathway modulation;
- Multi-target inhibition strategies.

#### 6.5.2. Methodological Priorities

- AI-assisted drug discovery pipelines;
- Advanced molecular simulation methods;
- Cryo-EM-guided structure determination;
- Patient-derived organoids;
- Functional genomic screening;
- Integrative multi-omics analyses.

#### 6.5.3. Translational Priorities

- Biomarker-driven clinical trials;
- Combination therapeutic approaches;
- Drug repurposing initiatives;
- Precision oncology platforms;
- ILC-specific therapeutic development programs.

The convergence of these approaches is expected to generate more effective and personalized treatment options for patients with ILC. As illustrated in Figure 6, the future of ILC drug discovery will likely involve integrating multi-omics profiling, artificial intelligence, structural biology, target validation, precision oncology, and clinical translation into a unified drug development framework.

## 7. Conclusions

Invasive lobular carcinoma is a biologically distinct subtype of breast cancer characterized by *CDH1* loss, unique signaling dependencies, and persistent clinical challenges related to endocrine resistance and metastatic progression. Advances in molecular profiling have revealed numerous actionable targets, including PI3K/AKT/mTOR, FGFR1, ROS1, SRC/FAK, YAP/TEAD, and estrogen receptor signaling networks, creating new opportunities for therapeutic intervention. Structure-based drug discovery has emerged as a powerful platform for exploiting these vulnerabilities by integrating structural biology, molecular docking, pharmacophore modeling, molecular dynamics simulations, free-energy calculations, and artificial intelligence-driven design strategies.

Despite ongoing challenges associated with tumor heterogeneity, resistance mechanisms, and limited ILC-specific clinical studies, recent technological advances are accelerating the discovery of novel therapeutic candidates. Particularly promising directions include targeting *CDH1*-loss-associated synthetic-lethal vulnerabilities, developing multi-target inhibitors, leveraging natural product-inspired scaffolds, and implementing precision oncology approaches guided by comprehensive molecular profiling.

Future success will depend on close integration of computational predictions with experimental validation using biologically relevant models, including organoids and patient-derived systems. The convergence of structure-based drug discovery, artificial intelligence, multi-omics technologies, and translational oncology is expected to significantly advance the development of personalized therapies for ILC, ultimately improving clinical outcomes for patients with this understudied yet clinically important breast cancer subtype.

## Figures and Tables

**Figure 1:**
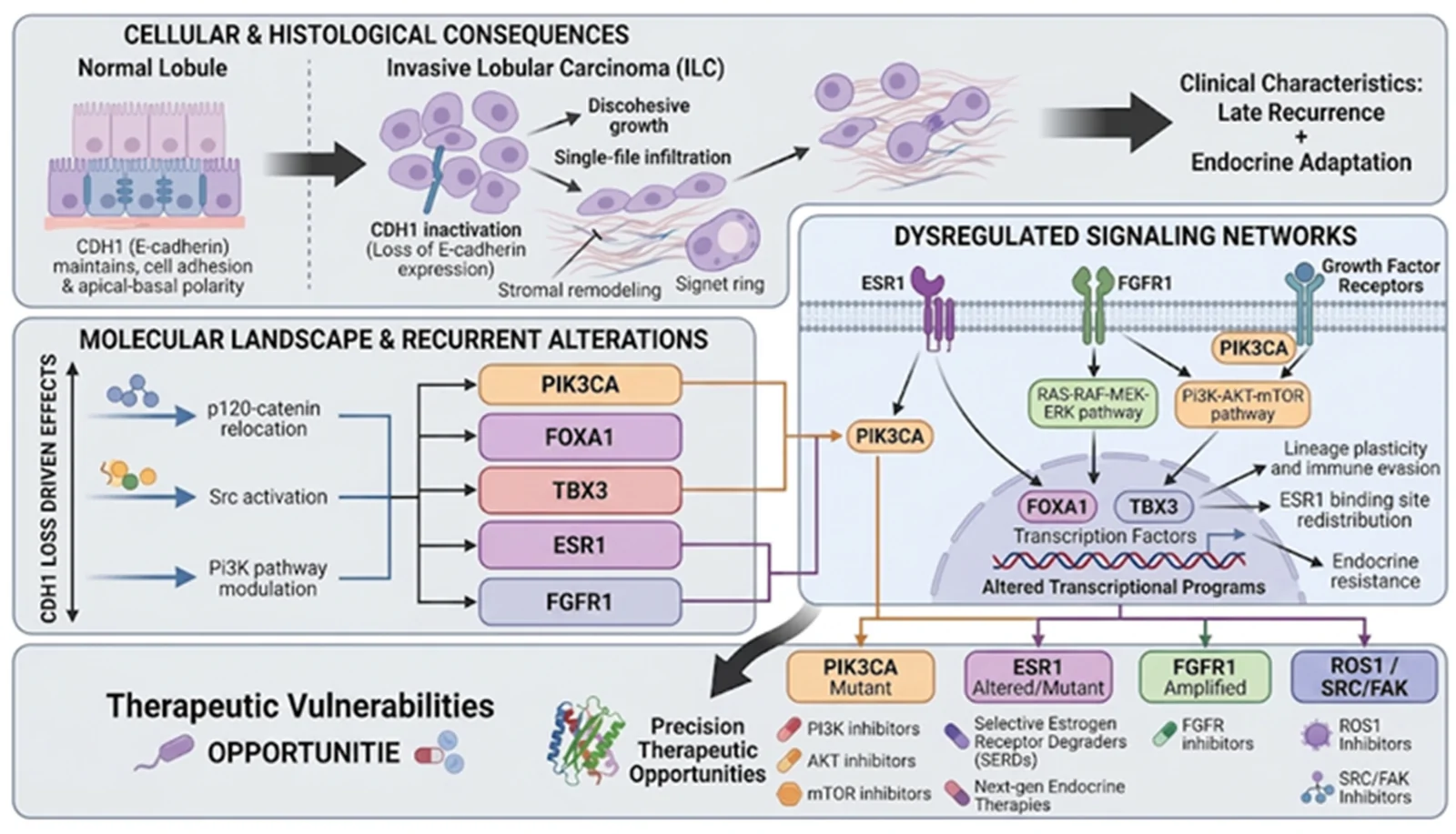
Integrated molecular landscape of invasive lobular carcinoma (ILC) illustrating the consequences of *CDH1* loss, recurrent genomic alterations, dysregulated signaling pathways, and associated therapeutic vulnerabilities.

**Figure 2:**
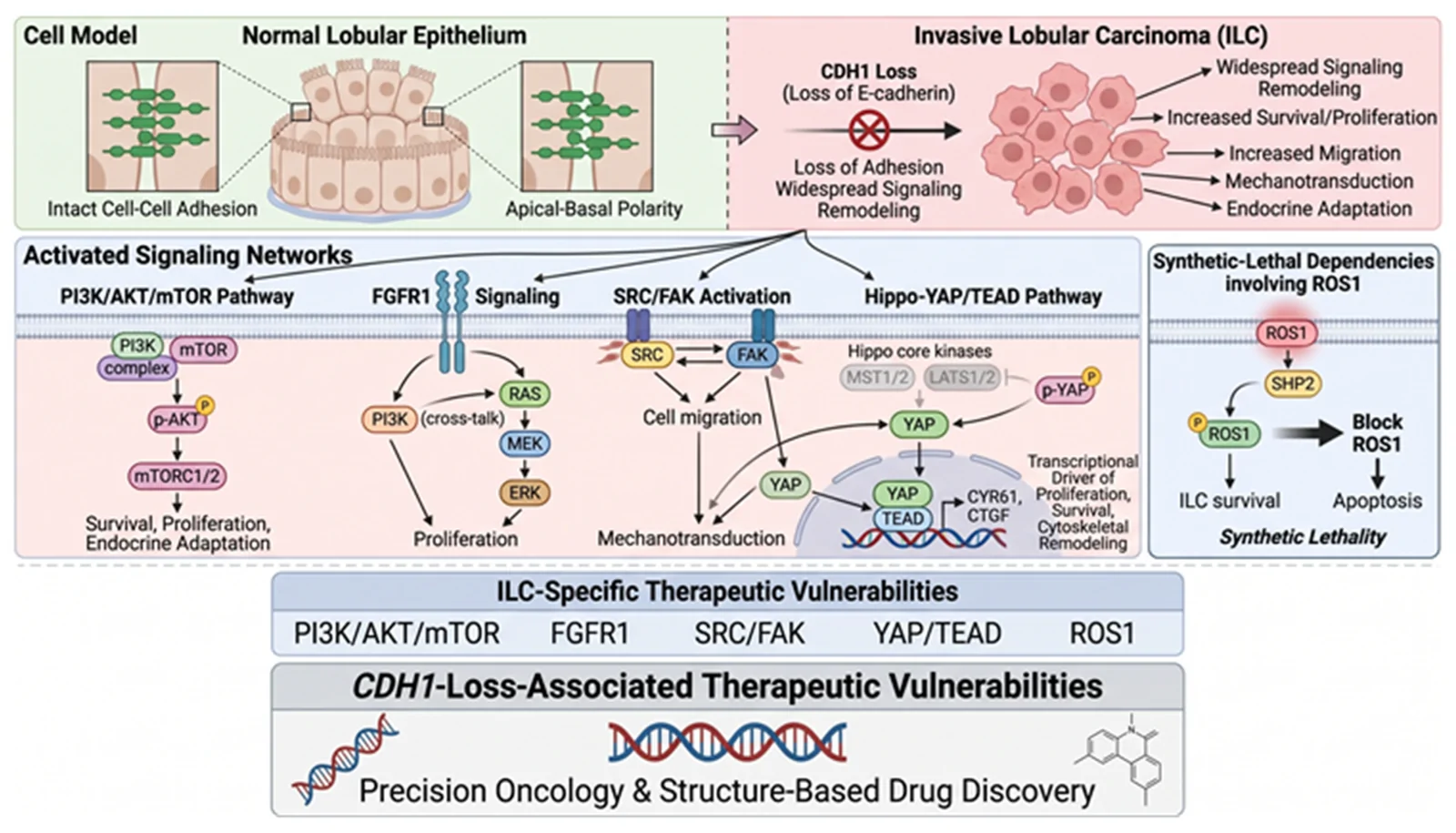
*CDH1*-loss-associated signaling dependencies and therapeutic vulnerabilities in invasive lobular carcinoma, highlighting key pathways involved in survival, proliferation, migration, mechanotransduction, endocrine adaptation, and synthetic-lethal interactions.

**Figure 3:**
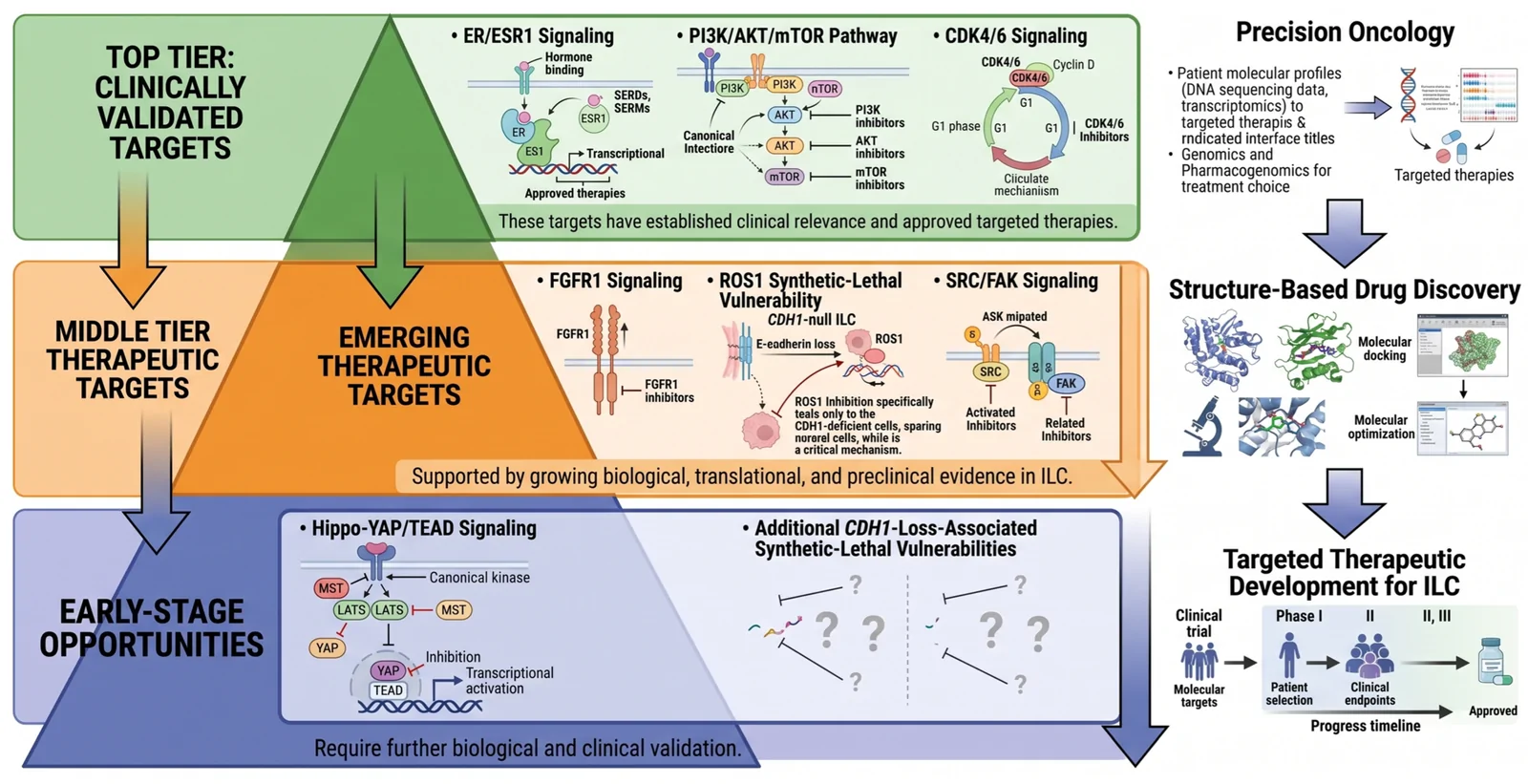
Evidence-based prioritization of therapeutic targets in invasive lobular carcinoma according to their current level of clinical, translational, and biological validation.

**Figure 4:**
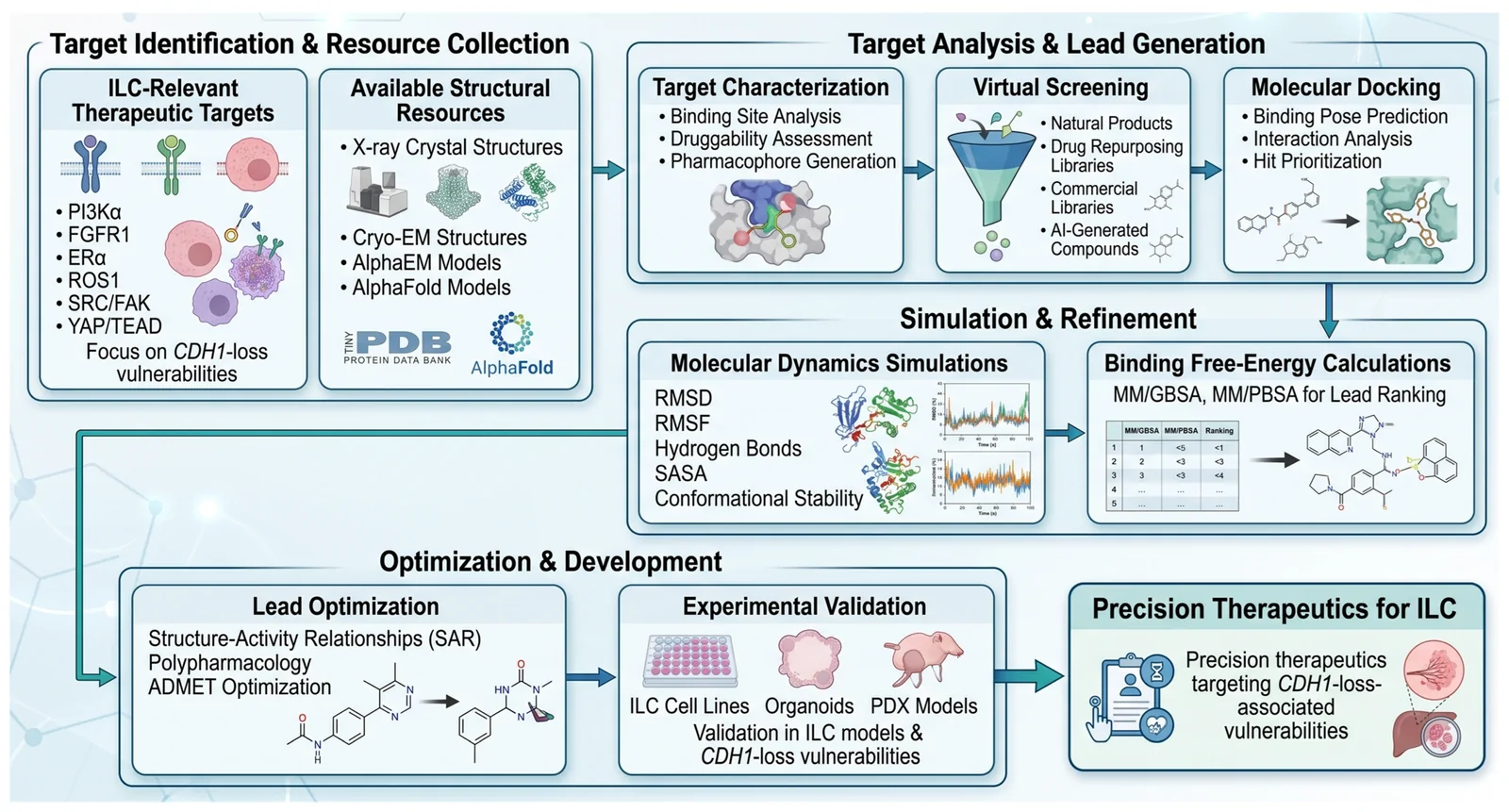
Structure-based drug discovery workflow for invasive lobular carcinoma, integrating target identification, structural characterization, virtual screening, molecular docking, molecular dynamics simulations, lead optimization, and experimental validation.

**Figure 5:**
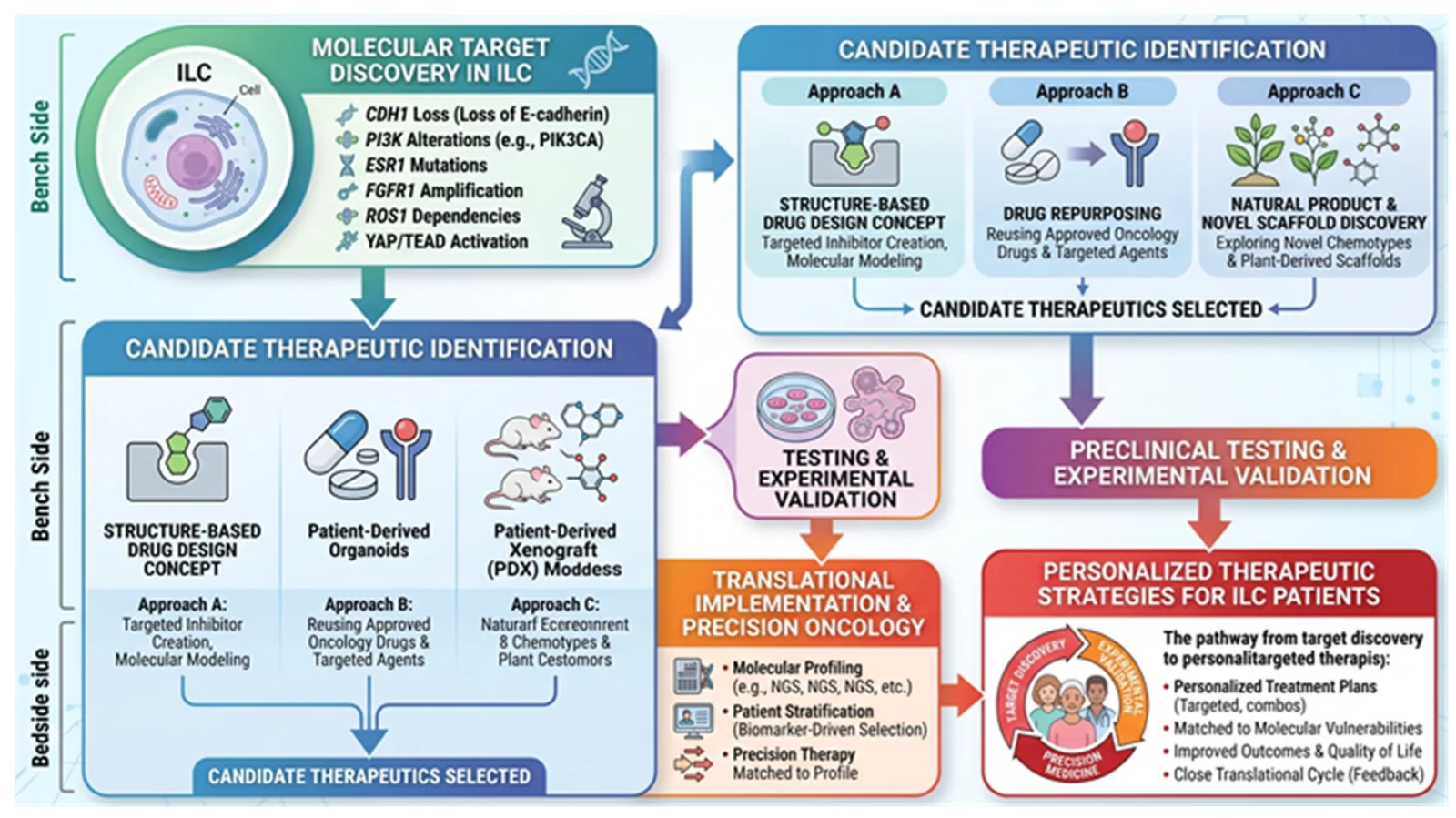
Translational framework linking therapeutic discovery approaches, preclinical validation, biomarker development, and precision oncology strategies in invasive lobular carcinoma.

**Figure 6:**
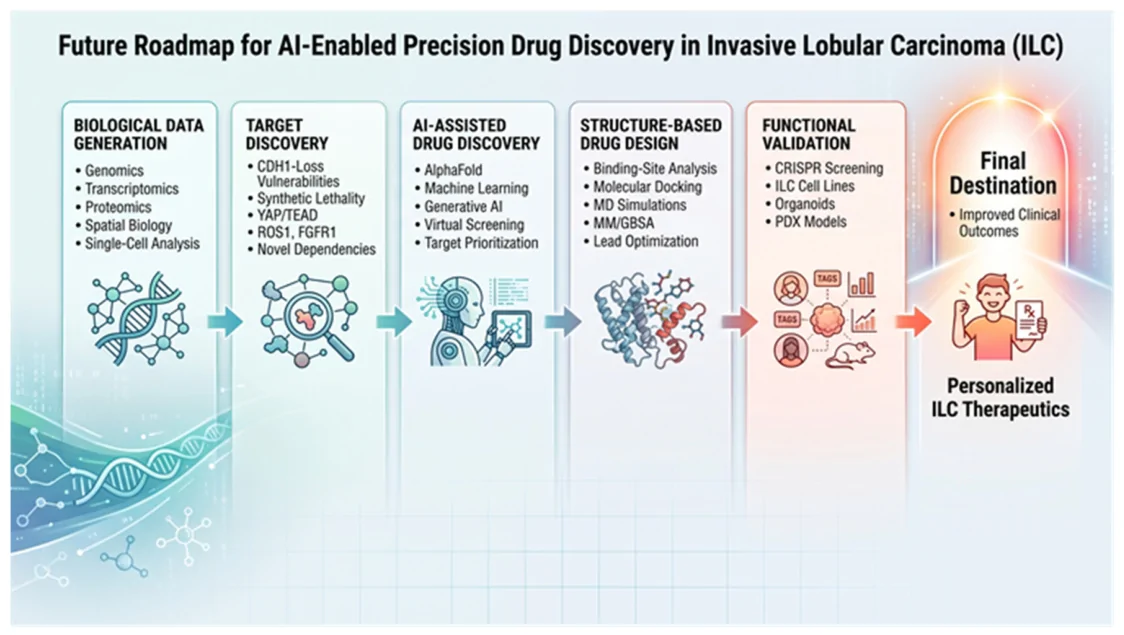
Future roadmap for AI-enabled precision drug discovery in invasive lobular carcinoma, illustrating the integration of multi-omics technologies, artificial intelligence, structural biology, functional validation, and precision oncology.

**Table 1:** Major genomic alterations in invasive lobular carcinoma and their therapeutic relevance.

Gene/ Alteration	Biological Function	Frequency in ILC	Functional Consequence	Therapeutic Relevance	Representative Therapeutic Strategies	Relative Enrichment in ILC vs. IDC	Reference(s)
*CDH1*	Encodes E-cadherin, a key mediator of epithelial cell–cell adhesion	~50–70% mutation; E-cadherin loss in >90% of ILC	Loss of cell adhesion, increased invasion, altered mechanotransduction, and synthetic lethal dependencies	Defining hallmark of ILC: source of targetable vulnerabilities	*ROS1* inhibition, SRC/FAK targeting, synthetic lethality-based therapies	Strongly enriched	[10,23,24,25,26]
*PIK3CA*	Catalytic subunit of PI3K	~40–50%	Constitutive PI3K/AKT/mTOR pathway activation	Driver of proliferation, survival, and endocrine resistance	PI3K inhibitors (e.g., alpelisib), AKT inhibitors, mTOR inhibitors	Common in both	[10,15,27,30]
*ERBB2* (HER2)	Receptor tyrosine kinase	~5–15% activating mutations	Enhanced HER2 signaling and growth promotion	Actionable oncogenic driver in selected ILC cases	Neratinib, tucatinib, trastuzumab-based therapies	Present in both, a subset of ILC	[10,15,18]
*ESR1*	Estrogen receptor α	More common in metastatic/endocrine-resistant disease	Ligand-independent ER signaling	Major mechanism of endocrine resistance	SERDs, SERMs, ER degraders, combination therapies	Common in advanced HR+ disease	[14,31,32]
*FOXA1*	Pioneer transcription factor regulating ER chromatin binding	~5–15%	Reprogramming of ER transcriptional networks	Associated with endocrine adaptation and resistance	Emerging target: indirect therapeutic modulation	Enriched	[15,17,18,33]
*TBX3*	Transcriptional regulator involved in development and differentiation	~10–15%	Altered transcriptional control and tumor progression	Potential biomarker of lobular tumor biology	Investigational target	Enriched	[10,15]
*PTEN*	Negative regulator of PI3K signaling	Low to moderate frequency	Hyperactivation of PI3K/AKT pathway	Contributes to therapeutic resistance	PI3K/AKT/mTOR pathway inhibitors	Common in multiple breast cancer subtypes	[11,13,15,27,30]
*FGFR1* *Amplification*	Receptor tyrosine kinase	~10–20%	Enhanced MAPK and PI3K signaling	Associated with aggressive disease and endocrine resistance	FGFR inhibitors (erdafitinib, futibatinib, pemigatinib)	Associated with endocrine-resistant HR+ disease	[32,34]
*CCND1* *Amplification*	Cell-cycle regulation through Cyclin D1	Variable	Increased cell-cycle progression	Supports CDK4/6-driven growth	CDK4/6 inhibitors	Common in HR+ breast cancers	[10,13,15]
*AKT1*	Key PI3K-pathway serine/threonine kinase	Low frequency	Constitutive survival signaling	Emerging therapeutic target	AKT inhibitors (capivasertib, ipatasertib)	Not clearly enriched; occurs in both ILC and IDC	[22,26,33]
*ERBB3* (HER3)	Co-receptor in HER signaling network	Low frequency	Activation of PI3K signaling	Potential contributor to endocrine resistance	HER3-targeted therapeutics	Not clearly enriched; occurs in both ILC and IDC	[10,15]
*NF1*	Negative regulator of RAS signaling	Occasional	Increased MAPK pathway activation	Potential resistance mechanism	MEK- or RAS-pathway-directed strategies	Not clearly enriched; occurs across multiple breast cancer subtypes	[10,15]

**Table 2:** Current and emerging druggable targets in invasive lobular carcinoma, associated signaling pathways, therapeutic rationale, representative inhibitors, and clinical development status.

Target	Associated Pathway	Biological Rationale in ILC	Representative Inhibitors/Agents	Clinical Development Status	Reference(s)
PI3Kα (*PIK3CA*)	PI3K/AKT/mTOR	Frequently mutated in ILC; promotes proliferation, survival, and endocrine resistance.	Alpelisib, Inavolisib, Taselisib	Approved/Advanced Clinical Development	[13,27,30,35,36]
AKT	PI3K/AKT/mTOR	Downstream mediator of PI3K signaling; contributes to resistance mechanisms.	Capivasertib, Ipatasertib	Approved/Advanced Clinical Development	[30,36]
mTOR	PI3K/AKT/mTOR	Central regulator of protein synthesis and cell growth	Everolimus, Temsirolimus	Approved/Clinical Use	[30,36]
FGFR1	FGFR/MAPK/PI3K	Amplified in endocrine-resistant and aggressive ILC; promotes tumor progression	Erdafitinib, Pemigatinib, Futibatinib, Infigratinib	Clinical Development	[32,37,38,39]
ERα (*ESR1*)	Estrogen Receptor Signaling	Major oncogenic driver in hormone receptor-positive ILC	Fulvestrant, Elacestrant, Tamoxifen	Approved	[14,31,40]
*Mutant ESR1*	Endocrine Resistance Pathway	Ligand-independent estrogen signaling in resistant disease	Elacestrant, Camizestrant, Imlunestrant	Clinical Development/Approved	[14,40]
CDK4/6	Cell Cycle Regulation	Supports proliferation downstream of ER signaling	Palbociclib, Ribociclib, Abemaciclib	Approved	[4,6,30]
HER2 (*ERBB2*)	HER Family Signaling	Activating mutations occur in a subset of ILC cases	Trastuzumab, Tucatinib, Neratinib, Trastuzumab Deruxtecan	Approved/Clinical Development	[18,28]
HER3 (*ERBB3*)	HER/PI3K Signaling	Contributes to adaptive resistance and survival signaling	Patritumab Deruxtecan	Clinical Development	[15,18]
ROS1	Synthetic-Lethal Signaling	Selective vulnerability associated with *CDH1* deficiency	Crizotinib, Entrectinib, Repotrectinib	Approved in Other Malignancies; Emerging in ILC	[19,41,42,43]
SRC	Cell Adhesion and Migration	Activated following E-cadherin loss; promotes invasion and metastasis	Dasatinib, Bosutinib, Saracatinib	Clinical Development	[28,44,45,46]
FAK (*PTK2*)	Focal Adhesion Signaling	Regulates migration, invasion, and mechanotransduction	Defactinib, GSK2256098	Clinical Development	[28,44,45]
YAP/TEAD	Hippo Signaling	Activated by loss of cell–cell adhesion and mechanosensing defects	VT3989, IK-930, TEAD Palmitoylation Inhibitors	Early Clinical Development	[14,47,48,49]
IGF1R	IGF Signaling	Enhanced signaling observed following E-cadherin loss	Linsitinib, Xentuzumab	Clinical Development	[18]
PARP	DNA Damage Repair	Potential vulnerability in selected genomically unstable tumors	Olaparib, Talazoparib	Approved in Appropriate Genetic Contexts	[13,15]
MEK/ERK	MAPK Pathway	Activated downstream of FGFR and other RTKs	Trametinib, Cobimetinib	Clinical Development	[37,38]
TEAD Protein–Protein Interaction Interface	Transcriptional Regulation	Emerging PPI target with increasing druggability evidence	VT3989, GQ1010, and related agents	Early Clinical Development	[48,49]

Abbreviations: ILC, invasive lobular carcinoma; ER, estrogen receptor; PI3K, phosphatidylinositol-3-kinase; AKT, protein kinase B; mTOR, mammalian target of rapamycin; *FGFR1*, fibroblast growth factor receptor 1; HER2, human epidermal growth factor receptor 2; FAK, focal adhesion kinase; PARP, poly(ADP-ribose) polymerase.

**Table 3:** Structural Resources and Structure-Based Drug Discovery Readiness of Major Therapeutic Targets in Invasive Lobular Carcinoma.

Target	Representative Structural Resource	Key Druggable Structural Feature	Structural Availability	SBDD Readiness	Reference(s)
PI3Kα (*PIK3CA*)	Multiple inhibitor-bound crystal structures	ATP-binding pocket and allosteric regions	Extensive	High	[64,65]
AKT	Active and inactive kinase structures	ATP-binding site and allosteric pockets	Extensive	High	[66]
mTOR	Cryo-EM and crystallographic structures	Catalytic kinase domain and FKBP12-rapamycin interface	Extensive	High	[66]
FGFR1	Multiple kinase-domain crystal structures	ATP-binding pocket and resistance-associated regions	Extensive	High	[66,67]
ERα (*ESR1*)	Ligand-bound receptor structures	Hormone-binding cavity and co-regulator interfaces	Extensive	High	[68]
HER2 (*ERBB2*)	Extracellular and kinase-domain structures	ATP-binding site and receptor dimerization interfaces	Extensive	High	[67]
HER3 (*ERBB3*)	Receptor ectodomain and kinase-domain models	Dimerization and signaling interfaces	Moderate	Moderate	[67]
ROS1	Inhibitor-bound kinase-domain structures	ATP-binding cavity and resistance mutation sites	Moderate	Moderate–High	[43]
SRC	Extensive kinase-domain structures	Catalytic pocket and SH2/SH3 regulatory domains	Extensive	Moderate–High	[69]
FAK (*PTK2*)	Kinase and FERM-domain structures	ATP-binding site and protein interaction interfaces	Extensive	Moderate–High	[45]
IGF1R	Kinase-domain structures	ATP-binding pocket and activation loop	Moderate	Moderate	[67]
PARP1	Multiple inhibitor-bound crystal structures	Catalytic NAD^+^ binding site	Extensive	High	[66]
TEAD Family Proteins	Structures containing a central lipid-binding pocket	Palmitoylation pocket and YAP-binding interface	Emerging	Moderate	[70,71]
YAP/TEAD Complex	Protein–protein interaction structures and computational models	Transcriptional complex interface	Emerging	Low–Moderate	[70,71]
AlphaFold-Predicted Targets	AI-predicted structural models	Enables structural analysis of poorly characterized proteins	Emerging	Discovery-stage	[22]

**Table 4:** Natural products and natural product-derived compounds reported to modulate ILC-relevant therapeutic targets and signaling pathways.

Natural Product/ Derivative	Natural Source	Major Molecular Target(s)	Associated Signaling Pathway	Reported Anticancer Mechanism	Translational Relevance to ILC	Evidence Level *	Reference(s)
Curcumin	*Curcuma longa* (Turmeric)	PI3K, AKT, mTOR, NF-κB	PI3K/AKT/mTOR	Induces apoptosis, suppresses proliferation, and survival signaling	Potential adjunct against PIK3CA-driven ILC and endocrine resistance	Breast cancer/Preclinical	[94,95,96,97,98,99]
Resveratrol	Grapes, berries, peanuts	PI3K, AKT, ERα, STAT3	PI3K/AKT and ER signaling	Anti-proliferative, pro-apoptotic, anti-metastatic effects	May modulate ER-positive ILC biology	Breast cancer/Preclinical	[94,95,96,97,100,101]
Quercetin	Fruits and vegetables	PI3K, EGFR, SRC	PI3K/AKT and SRC pathways	Cell-cycle arrest, apoptosis, and inhibition of invasion	Potential relevance for SRC-dependent ILC progression	Breast cancer/Preclinical	[95,96,97,102]
Genistein	Soybean-derived isoflavone	ERα, PI3K, AKT	Estrogen receptor signaling	Modulates ER activity and inhibits proliferation	Relevant to hormone receptor-positive ILC	Breast cancer/Preclinical	[95,96,103]
Epigallocatechin Gallate (EGCG)	Green tea (*Camellia sinensis*)	PI3K, AKT, HER2	PI3K/AKT and HER signaling	Anti-proliferative and anti-angiogenic activity	Potential utility in HER2- and PI3K-driven ILC	Breast cancer/Preclinical	[94,95,96,97,104]
Berberine	*Berberis* species	AKT, mTOR, AMPK	PI3K/AKT/mTOR	Suppresses growth and promotes apoptosis	Candidate scaffold for PI3K-targeted therapies	Pan-cancer/Preclinical	[95,96,105]
Luteolin	Celery, parsley, peppers	FGFR, PI3K, STAT3	FGFR and PI3K signaling	Anti-proliferative and anti-migratory effects	Potential relevance in FGFR1-amplified ILC	Pan-cancer/Preclinical	[96,106]
Apigenin	Chamomile, parsley	AKT, FAK, SRC	FAK/SRC signaling	Reduces migration and invasion	May interfere with adhesion-related pathways in CDH1-deficient tumors	Pan-cancer/Preclinical	[61,96]
Withaferin A	*Withania somnifera*	Vimentin, AKT, NF-κB	AKT signaling	Induces apoptosis and inhibits metastatic processes	Potential anti-invasive agent in ILC	Breast cancer/Preclinical	[60,90]
Parthenolide	*Tanacetum parthenium*	NF-κB, STAT3	Survival and inflammatory signaling	Promotes oxidative stress and apoptosis	Potential combination strategy for resistant disease	Pan-cancer/Preclinical	[107]
Paclitaxel	*Taxus* species (Natural product-derived drug)	Microtubules	Cell cycle regulation	Mitotic arrest and apoptosis	Clinically used in breast cancer treatment	Clinical	[62,93]
Vinblastine	*Catharanthus roseus*	Tubulin	Cell cycle regulation	Inhibits microtubule assembly	Established anticancer agent	Clinical	[62,93]
Camptothecin Derivatives (Irinotecan, Topotecan)	*Camptotheca acuminata*	Topoisomerase I	DNA damage response	DNA replication inhibition	Potential utility in selected advanced breast cancers	Clinical/Pan-cancer	[62,93]
Triptolide	*Tripterygium wilfordii*	YAP/TAZ, NF-κB	Hippo–YAP signaling	Suppresses transcriptional programs supporting tumor growth	Emerging candidate for YAP/TEAD-driven tumors	Pan-cancer/Preclinical	[108]
Natural Product-Inspired Molecular Hybrids	Synthetic derivatives of bioactive scaffolds	Multi-target (PI3K, FGFR1, SRC, ROS1)	Multiple pathways	Polypharmacology and improved target engagement	Attractive strategy for overcoming ILC heterogeneity and resistance	Drug Discovery/Computational	[63,97,109]

Abbreviations: ILC, invasive lobular carcinoma; PI3K, phosphatidylinositol-3-kinase; AKT, protein kinase B; mTOR, mammalian target of rapamycin; ERα, estrogen receptor alpha; FAK, focal adhesion kinase; SRC, proto-oncogene tyrosine-protein kinase Src; FGFR1, fibroblast growth factor receptor 1. * Evidence level categories indicate the highest level of available supporting evidence. ILC-specific = evidence generated directly in invasive lobular carcinoma models; Breast cancer = evidence reported in breast cancer systems but not specifically in ILC; Pan-cancer = evidence reported across multiple cancer types; Preclinical = in vitro and/or in vivo evidence only; Clinical = supported by human clinical evidence; Drug Discovery/Computational = supported primarily by computational, medicinal chemistry, or structure-based design studies.

**Table 5:** Major challenges affecting structure-based drug discovery in invasive lobular carcinoma and potential mitigation strategies.

Challenge Category	Specific Challenge	Impact on Drug Discovery	Potential Mitigation Strategies	Future Opportunities	Reference(s)
Limited ILC-Specific Research	Historically, fewer biological and translational studies compared with IDC	Restricted target validation and therapeutic development	Increased investment in ILC-focused research programs and collaborative consortia	Development of ILC-specific drug discovery pipelines	[4,5,6]
Tumor Heterogeneity	Interpatient variability in genomic and molecular alterations	Variable therapeutic responses and biomarker uncertainty	Multi-omics profiling and molecular stratification of patients	Precision oncology approaches	[10,15,116,117]
Endocrine Resistance	Acquired resistance to endocrine therapies through ESR1 mutations and pathway rewiring	Reduced long-term treatment efficacy	Combination therapies targeting PI3K, FGFR1, CDK4/6, and ER pathways	Personalized resistance-guided therapy	[14,29,30,112]
Incomplete Structural Information	Lack of high-resolution structures for some emerging targets and protein complexes	Limits effective structure-based design and screening	AlphaFold prediction, cryo-EM, homology modeling, integrative structural biology	Expansion of druggable target space	[22,58,59]
Protein–Protein Interaction (PPI) Druggability	Many ILC-relevant targets function through PPIs with shallow binding surfaces.	Difficult identification of high-affinity small-molecule modulators	Allosteric targeting, fragment-based design, covalent inhibitors, macrocycles	YAP/TEAD and transcriptional complex targeting	[48,49]
Synthetic-Lethal Target Validation	Limited functional validation of CDH1-loss-associated vulnerabilities	Delays translation of promising targets into therapies	CRISPR screening, functional genomics, organoid validation	Precision targeting of CDH1-deficient tumors	[19,41,118]
Resistance to Targeted Therapies	Adaptive pathway activation and signaling compensation	Reduced durability of therapeutic responses	Rational combination therapies and multi-target drug design	Improved long-term disease control	[30,112,113]
Translational Gap	Poor correlation between computational predictions and biological outcomes	High attrition of candidate compounds	Early experimental validation and iterative optimization	More predictive drug discovery workflows	[72,79,81]
Limited Experimental Models	Insufficient availability of representative ILC models	Weak biological validation of computational findings	Expanded use of organoids, patient-derived xenografts, and ex vivo models	Improved clinical relevance of preclinical studies	[26,54,115]
Natural Product Development Challenges	Poor solubility, bioavailability, and pharmacokinetic properties	Limits clinical translation of promising hits	Medicinal chemistry optimization and molecular hybridization	Natural product-inspired drug development	[93,94,97,111]
Data Integration Complexity	Increasing volume of genomic, proteomic, structural, and pharmacological data	Difficulty in prioritizing actionable targets	AI-driven analytics and multi-omics integration platforms	Systems-level precision oncology	[116,117,119,120,121]
Clinical Trial Limitations	Few ILC-specific clinical trials and patient cohorts	Delayed validation of emerging targets	Histology-specific trial designs and biomarker-driven enrollment	Accelerated therapeutic approval pathways	[4,6,116]
AI Model Reliability	Biases in training datasets and limited biological interpretability	Potentially misleading predictions	Explainable AI, experimental verification, and continuous model refinement	Robust AI-assisted drug discovery pipelines	[119,120,121]
Multi-Target Drug Design Complexity	Balancing potency, selectivity, and safety across multiple targets	Difficult lead optimization	Advanced computational design and polypharmacology strategies	FGFR1/PI3K, SRC/FAK, and ROS1-based multitarget therapies	[30,38,86,88]

Abbreviations: ILC, invasive lobular carcinoma; IDC, invasive ductal carcinoma; ER, estrogen receptor; PPI, protein–protein interaction; CRISPR, clustered regularly interspaced short palindromic repeats; AI, artificial intelligence; FGFR1, fibroblast growth factor receptor 1; PI3K, phosphatidylinositol-3-kinase.

## Data Availability

No new data were created or analyzed in this study. Data sharing is not applicable to this article.

## Abbreviations

The following abbreviations are used in this manuscript:

AACR	American Association for Cancer Research
AI	Artificial Intelligence
AKT	Protein Kinase B
CCND1	Cyclin D1
CDH1	Cadherin-1
CDK4/6	Cyclin-Dependent Kinases 4 and 6
CHPC	Centre for High-Performance Computing
Cryo-EM	Cryogenic Electron Microscopy
EGCG	Epigallocatechin Gallate
ER	Estrogen Receptor
ERBB2	Erb-B2 Receptor Tyrosine Kinase 2 (HER2)
ERBB3	Erb-B3 Receptor Tyrosine Kinase 3 (HER3)
ESR1	Estrogen Receptor 1
FAK	Focal Adhesion Kinase
FGFR1	Fibroblast Growth Factor Receptor 1
FOXA1	Forkhead Box A1
HER2	Human Epidermal Growth Factor Receptor 2
HER3	Human Epidermal Growth Factor Receptor 3
IDC	Invasive Ductal Carcinoma
IGC50	Half-Maximal Growth Inhibitory Concentration
IGF1R	Insulin-Like Growth Factor 1 Receptor
ILC	Invasive Lobular Carcinoma
MD	Molecular Dynamics
MM/GBSA	Molecular Mechanics/Generalized Born Surface Area
MM/PBSA	Molecular Mechanics/Poisson-Boltzmann Surface Area
mTOR	Mammalian Target of Rapamycin
NF1	Neurofibromin 1
NF-κB	Nuclear Factor Kappa B
PARP	Poly(ADP-Ribose) Polymerase
PDB	Protein Data Bank
PDX	Patient-Derived Xenograft
PI3K	Phosphatidylinositol-3-Kinase
PPI	Protein–Protein Interaction
PROTAC	Proteolysis-Targeting Chimera
PTEN	Phosphatase and Tensin Homolog
qPCR	Quantitative Polymerase Chain Reaction
RMSD	Root Mean Square Deviation
RMSF	Root Mean Square Fluctuation
ROS1	ROS Proto-Oncogene 1 Receptor Tyrosine Kinase
Rg	Radius of Gyration
SASA	Solvent Accessible Surface Area
SBDD	Structure-Based Drug Discovery
SERD	Selective Estrogen Receptor Degrader
SERM	Selective Estrogen Receptor Modulator
SRC	Proto-Oncogene Tyrosine-Protein Kinase Src
STAT3	Signal Transducer and Activator of Transcription 3
TBX3	T-Box Transcription Factor 3
TEAD	TEA Domain Transcription Factor
YAP	Yes-Associated Protein
TAZ	Transcriptional Co-Activator with PDZ-Binding Motif
